# Novel Findings in Teleost TRAF4, a Protein Acts as an Enhancer in TRIF and TRAF6 Mediated Antiviral and Inflammatory Signaling

**DOI:** 10.3389/fimmu.2022.944528

**Published:** 2022-07-11

**Authors:** Ying Chen, Ying Li, Peng Tian Li, Zi Hao Luo, Zi Ping Zhang, Yi Lei Wang, Peng Fei Zou

**Affiliations:** ^1^ Key Laboratory of Healthy Mariculture for the East China Sea, Ministry of Agriculture and Rural Affairs, Ornamental Aquarium Engineering Research Centre in University of Fujian Province, Fisheries College, Jimei University, Xiamen, China; ^2^ Key Laboratory of Estuarine Ecological Security and Environmental Health, Tan Kah Kee College, Xiamen University, Zhangzhou, China; ^3^ State Key Laboratory of Large Yellow Croaker Breeding, Ningde Fufa Fisheries Company Limited, Ningde, China; ^4^ College of Marine Science, Fujian Agriculture and Forestry University, Fuzhou, China

**Keywords:** TRAF4, TRIF, TRAF6, IRF3, IRF7, large yellow croaker

## Abstract

Tumor necrosis factor receptor-associated factors (TRAFs) are important adaptor molecules that play important roles in host immune regulation and inflammatory responses. Compared to other members of TRAFs, the function of TRAF4 in vertebrate immunity remains unclear, especially in teleosts. In the present study, TRAF4 ortholog was cloned and identified in large yellow croaker (*Larimichthys crocea*), named as *Lc-TRAF4*. The open reading frame (ORF) of *Lc*-*TRAF4* is 1,413 bp and encodes a protein of 470 amino acids (aa), which is consisted of a RING finger domain, two zinc finger domains, and a MATH domain. The genome organization of *Lc*-*TRAF4* is conserved in teleosts, amphibians, birds, and mammals, with 7 exons and 6 introns. Quantitative real-time PCR analysis revealed that *Lc*-*TRAF4* was broadly distributed in various organs/tissues of healthy large yellow croakers and could be significantly up-regulated in the gill, intestine, spleen, head kidney, and blood under poly I:C, LPS, PGN, and *Pseudomonas plecoglossicida* stimulations. Notably, luciferase assays showed that overexpression of *Lc*-TRAF4 could significantly induce the activation of IRF3, IRF7, and type I IFN promoters, with the RING finger and zinc finger domains function importantly in such promoter activation. Confocal microscopy revealed that *Lc*-TRAF4 is located in the cytoplasm, whereas the deletion of the RING finger, zinc finger or MATH domain showed little effect on the subcellular localization of *Lc*-TRAF4. Interestingly, *Lc*-TRAF4 overexpression could significantly enhance *Lc*-TRIF and *Lc*-TRAF6 medicated IRF3 and IRF7 promoter activation. In addition, co-expression of *Lc*-TRAF4 with *Lc*-TRIF or *Lc*-TRAF6 could significantly induce the expression of antiviral and inflammation-related genes, including *IRF3*, *IRF7*, *ISG15*, *ISG56*, *Mx*, *RSAD2*, *TNF-α*, and *IL-1β* compared to the only overexpression of *Lc*-TRAF4, *Lc*-TRIF or *Lc*-TRAF6. These results collectively imply that *Lc*-TRAF4 functions as an enhancer in *Lc*-TRIF and *Lc*-TRAF6 mediated antiviral and inflammatory signaling.

## Introduction

It is well known that the immune system of teleosts is composed of an innate immune system and an acquired immune system (1). The innate immune system, which acts as the first line of defense against pathogen invasion, plays an important role in host immune response (2). The innate immune system mainly relies on pattern recognition receptors (PRRs) to recognize pathogen-associated molecular patterns (PAMPs) and activate adaptor proteins, which then induce the production of interferons (IFNs), chemokines, inflammatory cytokines, and even initiate cell-death pathways to eliminate pathogen-infected or damaged cells (3–6). Five types of PRRs have been reported, including toll-like receptors (TLRs), retinoic acid-inducible gene I (RIG-I)-like receptors (RLRs), nucleotide-binding oligomerization domain (NOD)-like receptors (NLRs), C-type lectin receptors (CLRs), and absent in melanoma 2 (AIM2)-like receptors (ALRs) (4, 7).

Tumor necrosis factor receptor-associated factors (TRAFs) are a group of cytoplasmic adaptor proteins, which play important roles downstream of TLRs, RLRs, and NLRs mediated signaling pathways and are involved in host innate and adaptive immune responses (8, 9). Mammalian TRAF family is composed of seven members (TRAF1-TRAF7) (10). Except for TRAF7, all the members of the TRAF family contain a conserved C-terminal TRAF domain, which is consisted of TRAF-N and MATH domains (9, 11). In addition, except for TRAF1, all TRAF family members contain a RING finger domain at their N-terminal, followed by several zinc finger domains (8). TRAFs are adaptor proteins that mediate a variety of immune-related signaling pathways (12), which regulate host immune response and inflammation by inducing the activation of transcription factors such as nuclear transcription factor-κB (NF-κB), mitogen-activated protein kinase (MAPK), and interferon regulatory factors (IRFs) (9, 10, 13).

TRAF4, a member of the TRAF family, was first found in lymph node tissues of metastatic human breast cancer (14, 15). Studies in mammals have shown that TRAF4 can directly or indirectly interact with other molecules to regulate NF-κB, c-Jun N-terminal kinase (JNK), and MAPK signaling pathways and then participate in immune responses (11, 16). It was also found that TRAF4 is associated with p47phox, a component of cytosolic NADPH oxidase, and physically interacts and functionally counteracts with TRAF6 and TIR domain-containing adaptor-inducing IFN-β (TRIF) that critically regulate TLR-mediated signaling (17). Other studies have shown that TRAF4 and TRAF6 have the same TRAF binding site on NF-κB activator 1 (Act1), thereby inhibiting IL-17 signaling transduction (18).

Compared to some important progress in mammals, little is known about the function of TRAF4 in teleosts. In recent years, orthologs of TRAF4 have been cloned and identified in teleosts, including *Epinephelus coioides* (19), *Danio rerio* (20), and *Cynoglossus semilaevis* (21). The expression level of *TRAF4* was significantly upregulated under infection of *Cryptocaryon irritans* and red-spotted grouper nervous necrosis virus (RGNNV) in grouper (*E. coioides*). In addition, overexpression of EcTRAF4 decreased the expression of IFN-related molecules and proinflammatory factors (19). Another report in *C. semilaevis* also revealed that the mRNA expression level of TRAF4 was significantly up-regulated in response to *Vibrio harveyi* infection (21). These results suggest that TRAF4 may play important roles in host immune responses in teleosts, however, the signaling pathway that TRAF4 mediated and the adaptors that associated still remain unknown.

Large yellow croaker (*Larimichthys crocea*) is an important economic mariculture fish in the eastern and southern coastal areas of China (22, 23). In recent years, with the expansion of cultivation scale and deterioration of cultivation conditions, diseases frequently occurred in the cultivation process, including various diseases caused by parasites, bacteria, and viruses, which brought serious losses to the large yellow croaker industry (24–26). It is thus of great importance to understand the recognition of the pathogens and also the immune defense mechanisms that associated for further disease control and prevention of the fish. In the present study, an ortholog of TRAF4 was cloned and identified in large yellow croaker. The genome organization, subcellular localization, expression patterns of *TRAF4* in various organs/tissues as well as stimulation under various PAMPs or bacterial pathogen infection, and the induction of TRAF4 in IRF3, IRF7, and type I IFN promoter activation were also determined. Notably, the present study revealed the association of TRAF4 with TRIF and TRAF6 in IRF3 and IRF7 promoter activation, as well as their possible association in the induction of downstream immune-related molecules, including IRF3, IRF7, IFN-stimulating gene 15 (ISG15), ISG56, interferon-induced GTP-binding protein Mx (Mx), radical S-adenosyl methionine domain containing 2 (RSAD2), interleukin-1β (IL-1β), and tumor necrosis factor-α (TNF-α). These findings provide a new and essential perspective on understanding the roles of TRAF4 in host immune-related signaling pathway in teleosts.

## Materials and Methods

### Experimental Fish, Cell Lines, and Transfection

Large yellow croakers (18.0 ± 1.5 cm in length and 60.0 ± 15.0 g in weight) were purchased from Ningde Fufa Fishing Co., Ltd. The fish were kept in a 25°C inflatable seawater tank and fed with commercial feed for two weeks before being used for subsequent experiments as our previous reports (10, 27). The fish used in the present study were conducted according to the guidelines of the Animal Administration and Ethics Committee of Jimei University (Permit No. 2021-4).

HEK 293T cells were purchased from the Chinese Typical Culture Preservation Center and cultured in Dulbecco’s Modified Eagle Medium (DMEM) supplemented with 10% fetal bovine serum (FBS, Invitrogen-Gibco), 100 U/mL penicillin (P) and streptomycin (S). The cells were cultured in a 5% CO_2_ incubator at 37°C (10, 27). Large yellow croaker muscle cells (LYCMS) were maintained in L15 medium (Boster, USA) supplemented with 10% fetal bovine serum and 100 U/mL penicillin and streptomycin at 25°C. The transfection of the plasmids into the cells mentioned above was performed by using Lipofectamine^®^ 3000 (Invitrogen, Carlsbad, CA) according to the manufacturer’s instructions.

### Immune Challenge

To understand the tissue distribution of *TRAF4* in large yellow croakers, six healthy fish were randomly selected and anesthetized with 0.01% eugenol, then organs/tissues including gill, liver, spleen, head kidney, trunk kidney, intestine, heart, brain, skin, and muscle were dissected and stored in RNAlater, and blood was collected and stored in Trizol for subsequent total RNA extraction.

To study the expression patterns of *TRAF4* in large yellow croakers under different immune stimulations, the fish were divided into five groups (with each group contained 50 fish) and injected with different pathogen-associated molecular patterns (PAMPs). In brief, each fish in challenge groups was intraperitoneally injected with 100 μL suspension of *Pseudomonas plecoglossicida* (5×10^5^ CFU/mL), polyinosinic-poly-cytidylic acid potassium salt (poly I:C) (P9582, Sigma, 1 mg/mL), lipopolysaccharides (LPS) (L3024, Sigma, from *Escherichia coli* O111:B4, 0.5 mg/mL), and peptidoglycan (PGN) (69554, from Bacillus subtilis, Sigma, 1 mg/mL), respectively. The control group was injected with 100 µL PBS solution (27). Six fish were randomly selected from each group at 6, 12, and 24 h post-injection (hpi) and anaesthetized as described above, different organs/tissues such as gill, intestine, spleen, head kidney, and blood were collected for RNA extraction experiment.

### RNA Extraction, Gene Cloning, and Plasmids Construction

Total RNA was extracted from the above tissue/organ samples using Eastep™ Super Total RNA Extraction Kit (Promega) according to the manufacturer’s instructions. The quality and concentration of RNA were determined by agarose gel electrophoresis and OD260/280 analysis. 1 μg total RNA was taken from each sample and digested with RNase free DNase I (Thermo Scientific™), then RevertAid First Strand cDNA Synthesis Kit (#K1622, Thermo Scientific™) was used to reverse transcribe the RNA into cDNA, followed by storing in a refrigerator at -80°C for subsequent gene cloning or quantitative real-time PCR analysis (qRT-PCR).

Based on *TRAF4* transcriptome data of large yellow croaker (GenBank Accession No. XM_010749366.3), the full-length ORF of large yellow croaker *TRAF4* was amplified by using specific primers. The amplification conditions were as follows: 95°C denatured 3 min, 35 cycles of denaturation at 95°C, annealing at 56°C for 30 s, extension at 72°C for 2 min. The confirmed ORF sequence of *TRAF4* was inserted into pTurboGFP-N vector to construct GFP fluorescent tracer plasmid and pcDNA3.1/myc-His (−) A vector for subsequent eukaryotic overexpression analysis, which were verified by sequencing analysis, respectively. Primers used in the present study are shown in **Table 1**.

**Table 1:** Primer sequences used in the present study.

Primer Name	Accession No.	Sequence (5’-3’)	Application
*Lc*-TRAF4-F	XM_010749366.3	ATGCCCGGGTTTGATTACAA	*Lc*-TRAF4 ORF cloning
*Lc*-TRAF4-R		AGCCATTATCTTCTGGGGAATCTCT	
pcDNA3.1-TRAF4-F	ON246166	CCGCTCGAGCGATGGCTCCCGGGTTTGATTACAA	pcDNA3.1-TRAF4
pcDNA3.1-TRAF4-R		GGGGTACCAGCCATTATCTTCTGGGGAATCTCT	
pc3.1-TRAF4-ΔRING-F	ON246166	CCGCTCGAGCGATGGCCAAGATCTACCCAGACC	pc3.1-TRAF4-ΔRING
pc3.1-TRAF4-ΔRING-R		GGGGTACCAGCCATTATCTTCTGGGGAATCTCT	
pc3.1-TRAF4-ΔRING/zinc1-F	ON246166	CCGCTCGAGCGATGCAGGAGAGTGTTTACTGTGAGA	pc3.1-TRAF4-ΔRING/zinc 1
pc3.1-TRAF4-ΔRING/zinc1-R		GGGGTACCAGCCATTATCTTCTGGGGAATCTCT	
pc3.1-TRAF4-MATH-F	ON246166	CCGCTCGAGCGATGACCAAGTCTCACCTGACCATG	pc3.1-TRAF-MATH
pc3.1-TRAF4-MATH-R		GGGGTACCAGCCATTATCTTCTGGGGAATCTCT	
pc3.1-TRAF4-ΔMATH-F	ON246166	CCGCTCGAGCGATGCCCGGGTTTGATTACAAG	pc3.1-TRAF4-ΔMATH
pc3.1-TRAF4-ΔMATH-R		GGGGTACCTGACAGCTCCTCCATCTCCCTC	
pTurbo-TRAF4-F	ON246166	CCGCTCGAGATGGCTCCCGGGTTTGATTACAA	pTurbo-TRAF4-GFP
pTurbo-TRAF4-R		GGGGTACCGTAGCCATTATCTTCTGGGGAATCTCT	
pTurbo-TRAF4-ΔRING-F	ON246166	CCGCTCGAGATGGCCAAGATCTACCCAGACC	pTurbo-TRAF4-ΔRING-GFP
pTurbo-TRAF4-ΔRING-R		GGGGTACCGTAGCCATTATCTTCTGGGGAATCTCT	
pTurbo-TRAF4-ΔRING/zinc1-F	ON246166	CCGCTCGAGATGCAGGAGAGTGTTTACTGTGAGA	pTurbo-TRAF4-ΔRING/zinc 1-GFP
pTurbo-TRAF4-ΔRING/zinc1-R		GGGGTACCGTAGCCATTATCTTCTGGGGAATCTCT
pTurbo-TRAF4-MATH-F	ON246166	CCGCTCGAGATGACCAAGTCTCACCTGACCATG	pTurbo-TRAF4-MATH-GFP
pTurbo-TRAF4-MATH-R		GGGGTACCGTAGCCATTATCTTCTGGGGAATCTCT	
pTurbo-TRAF4-ΔMATH-F	ON246166	CCGCTCGAGATGCCCGGGTTTGATTACAAG	pTurbo-TRAF4-ΔMATH-GFP
pTurbo-TRAF4-ΔMATH-R		GGGGTACCGTTGACAGCTCCTCCATCTCCCTC	
qTRAF4-F	ON246166	CATCAGTACCACTGCCCTCG	qRT-PCR
qTRAF4-R		AGATGACGACCGATAGCCAGT	
qβ-actin-F	FJ936563.1	CCTTCACCACCACAGCCGAG	qRT-PCR
qβ-actin-R		ATTCCGCAAGATTCCATACCGA	
qIRF3-F	NM_001303387.1	AAGATGGGCGATGGTTTGG	qRT-PCR
qIRF3-R		GCTCTATGGGCTGTCTGCTACTG	
qIRF7-F	NM_001303350.1	ATGGGCAGTAGCAAGTGGTAAA	qRT-PCR
qIRF7-R		ACTCTGTGGGCGAGTTGTAGAT	
qISG15-F	MH280016.1	GCGATGACTCTCAGTGTATCAGT	qRT-PCR
qISG15-R		TGACAGTCTCCTCCGGTTTG	
qISG56-F	EU200362.2	GCGCGATAGAAACAGGTCAAT	qRT-PCR
qISG56-R		TGCCAGGAAGGCCTCTATTTC	
qMx-F	XM_019274379.2	AGGATAAAATGGCGGGAAGT	qRT-PCR
qMx-R		AAAGCCTCTGTGGTTGCTATGT	
qRSAD2-F	MF153478.1	CCCAAGTGTCAGCATCGTCA	qRT-PCR
qRSAD2-R		TGCGAATCTTGTAAAGGCAATC	
qIL-1β-F	KP057877.1	TCACTTGAACATGGCGACCTA	qRT-PCR
qIL-1β-R		GGTCGTTGTCCCCATCCCTA	
qTNFα-F	EF165623.1	GCGTCGTTCAGAGTCTCCTGC	qRT-PCR
qTNFα-R		CGTTGTACCACCCGTGTCCC	

### Bioinformatics Analysis

Protein prediction was carried out using the software at ExPASy Bioinformatics Resource Portal (http://www.expasy.org/proteomics), with conserved domain structures detected by using the Conserved Domain Database (CDD) on NCBI (http://www.ncbi.nlm.nih.gov/cdd) (28). Vertebrate TRAF4 protein sequences were searched using the Basic Local Alignment Search Tool (BLAST) analysis tool of the National Center for Biotechnology Information website (NCBI, http://www.ncbi.nlm.nih.gov/blast). Amino acid multiple alignments were performed using the Clustal X program and the results were edited using the GeneDoc program. The phylogenetic analysis was conducted using the neighbor-joining method in MEGA 7 (29).

### qRT-PCR Analysis

The above cDNA was used as a template, and the volume of qPCR amplification was 10 µL, including 2×SYBR Green Master Mix 5 µL, forward primer 0.5 µL (4 mM), reverse primer 0.5 µL (4 mM), cDNA 1 µL (100 ng/µL), and nuclease-free water 3 µL.

qRT-PCR was performed using Go Taq^®^ qPCR Master Mix (Promega, Madison, USA) on the Roche LightCycler^®^480 II quantitative real-time detection system (Roche, Switzerland). The PCR amplification conditions were as follows: 95°C for 5 min, followed by 40 cycles at 95°C for 20 s, 58°C for 15 s, and 72°C for 15 s, with a final extension at 72°C for 10 min, and then the reaction temperature was increased from 65°C to 95°C at a rate of 0.5°C per second, and the melting curve was analyzed. All the reactions were carried out in a 384-well plate and their mean values were recorded. The relative expression of the target gene was normalized to the expression of *β-actin*, which has been proved to be a reliable reference gene and widely used for gene expression analysis in large yellow croaker (10, 27), and then analyzed using a comparative Ct method (2^-ΔΔCt^) (30). All primers used for qRT-PCR were listed in **Table 1**.

### Luciferase Activity Assays

To determine the effect of large yellow croaker TRAF4 on IRF3, IRF7, and type I IFN promoter activation, HEK 293T cells were transiently transfected with 100 ng pGL4-IRF3-pro (Chinese invention patent number: ZL201710457836.8), pGL4-IRF7-pro (Chinese invention patent number: ZL201710457820.7) or pGL4-IFN1-pro (Chinese invention patent application number: 201710456729.3), 10 ng of pRL-TK (Promega, Madison, WI) together with increasing concentrations of pcDNA3.1-TRAF4 (10, 50, 100 or 200 ng) or pcDNA3.1 empty vector with the same concentrations for luciferase activity assays.

To determine the effect of different domains of TRAF4 on IRF3, IRF7, and type I IFN promoter activation, HEK 293T cells were transiently transfected with 100 ng pGL4-IRF3-pro, pGL4-IRF7-pro or pGL4-IFN1-pro, 10 ng pRL-TK together with 100 ng full-length TRAF4 and its truncated forms including TRAF4-ΔRING (residues 63-470), TRAF4-ΔRING/zinc1 (residues 161-470), TRAF4-MATH (residues 273-470), and TRAF4-ΔMATH (residues 1-303) for luciferase activity assays.

To investigate the association between large yellow croaker TRAF4 and TRIF or TRAF6, HEK 293T cells were seeded overnight in a 24-well plate with 1 × 10^5^ cells per well. The next day, the cells were transiently transfected with 100 ng pGL4-IRF3-pro or pGL4-IRF7-pro, 10 ng of pRL-TK together with 100 ng pcDNA3.1-TRAF4 and pcDNA3.1-TRIF or pcDNA3.1-TRAF6 alone or in combination of two, with the total amount of transfected plasmids balanced with the pcDNA3.1 empty vector.

At 24 hours post-transfection (hpt), the transfected cells described above were collected and lysed for subsequent luciferase activity assays using Dual-Luciferase Reporter System (Promega), with luciferase activity measured by a Promega GloMax^®^ 20/20 luminometer according to the manufacturer’s instructions. All data were normalized to *Renilla* luciferase activity and expressed as fold relative to the control group.

### Confocal Microscopy

To detect the subcellular localization of TRAF4 and truncated forms of TRAF4 in large yellow croaker, HEK 293T cells were cultured on sterilized coverslips in 6-well plates overnight (2 × 10^5^ cells per well). On the second day, 5 µg plasmids of pTurbo-TRAF4-GFP, pTurbo-TRAF4-ΔRING-GFP, pTurbo-TRAF4-ΔRING/zinc1-GFP, pTurbo-TRAF4-MATH-GFP, pTurbo-TRAF4-ΔMATH-GFP, and pTurboGFP-N (vector control) were transfected into HEK 293T cells with Lipofectamine^®^ 3000, respectively. At 24 hpt, the transfected cells growing on the coverslips were washed with PBS, fixed with 4% paraformaldehyde at room temperature for 10 min, and then permeated with Triton X-100. The cells on the coverslips were washed with PBS and sealed with one drop of the mounting medium (VECTASHIELDR Hard Set™ Mounting Medium with DAPI, Vector Laboratories, CA). Finally, the cells were examined and photographed under a confocal microscope (Laika, Germany).

### Effects of TRAF4 With TRIF and TRAF6 on Antiviral and Inflammation-Related Genes Expression

To determine the effects of large yellow croaker TRAF4 with TRIF and TRAF6 on the induction of downstream antiviral and inflammation-related genes expression, LYCMS cells were seeded on 6-well plates at a density of 2 × 10^5^ cells per well, and then cells were co-transfected with 2.5 μg pcDNA3.1-TRAF4 together with 2.5 μg pcDNA3.1-TRIF or pcDNA3.1-TRAF6 alone or in a combination of two, with the total amount of transfected plasmids balanced with the pcDNA3.1 empty vector. At 48 hpt, the cells were collected for total RNA extraction and cDNA synthesis. Subsequently, qRT-PCR was performed to detect the mRNA expression levels of antiviral and inflammation-related genes, including *IRF3*, *IRF7*, *ISG15*, *ISG56*, *Mx*, *RSAD2*, *IL-1β*, and *TNF-α*. All primers used for qRT-PCR are shown in **Table 1**.

### Statistical Analysis

Statistical analysis was conducted using one-way analysis of variance (ANOVA), followed by Duncan’s multiple range test using SPSS version 25, with different superscripts indicating statistically different (*P* < 0.05), * *P* < 0.05, and ** *P* < 0.01 considered statistically significant, respectively.

## Results

### Identification of Teleost TRAF4 Ortholog in Large Yellow Croaker

To study the role of TRAF4 in host immunity of large yellow croaker, the cDNA of *TRAF4* was cloned from the spleen of the fish and named as *Lc-TRAF4* (GenBank accession No. ON246166). The identified ORF of *Lc-TRAF4* consists of 1,413 nucleotides, encoding a protein of 470 amino acids (aa). Based on the analysis of conserved domains by using NCBI CDD, it was found that *Lc*-TRAF4 contains a RING finger domain (15-59 aa), two zinc finger domains (102-156 aa, and 210-269 aa), and a MATH domain (308-463 aa) (**Figure S1**).

The amino acid sequence of *Lc*-TRAF4 was compared with TRAF4 of other vertebrates, including grouper (*E. coioides*), medaka (*Oryzias latipes*), rainbow trout (*Oncorhynchus mykiss*), fugu (*T. rubripes*), zebrafish (*Danio rerio*), Atlantic salmon (*Salmo salar*), channel catfish (*Ictalurus punetaus*), African clawed frog (*Xenopus laevis*), mouse (*Mus musculus*), and human (*Homo sapiens*) (**Figure S1**), which revealed a relatively conservative RING finger domain, zinc finger domains, and MATH domain in the species that mentioned. Additionally, the amino acid sequence of *Lc*-TRAF4 had a similarity of 99% with the TRAF4 of grouper, medaka, rainbow trout, and fugu, 98% with zebrafish, and 97% with channel catfish. Compared with TRAF4 of amphibians and mammals, the similarity level decreased, with a similarity of 92% to African clawed frog and 90% to mouse and human (**Table 2**), indicating a high degree of sequence conservation across vertebrate TRAF4 proteins.

**Table 2:** Amino acid identity and similarity among *Lc*-TRAF4 and TRAF4 from other vertebrates.

Common Name	Scientific Name	Accession No.	Length (aa)	Identity	Similarity
Grouper	*Epinephelus coioides*	XP_021443644.1	470	99%	99%
Medaka	*Oryzias latipes*	XP_021443644.1	470	98%	99%
Rainbow trout	*Oncorhynchus mykiss*	XP_021443644.1	470	95%	99%
Fugu	*Takifugu rubripes*	XP_021443644.1	470	97%	99%
Zebrafish	*Danio rerio*	CAD89005.1	470	95%	98%
Channel catfish	*Ictalurus punetaus*	XP_017346543.1	470	92%	97%
African clawed frog	*Xenopus laevis*	NP_001087501.1	470	80%	92%
Mouse	*Mus musculus*	NP_033449.2	470	76%	90%
Human	*Homo sapiens*	NP_004286.2	470	77%	90%

**Figure 1:**
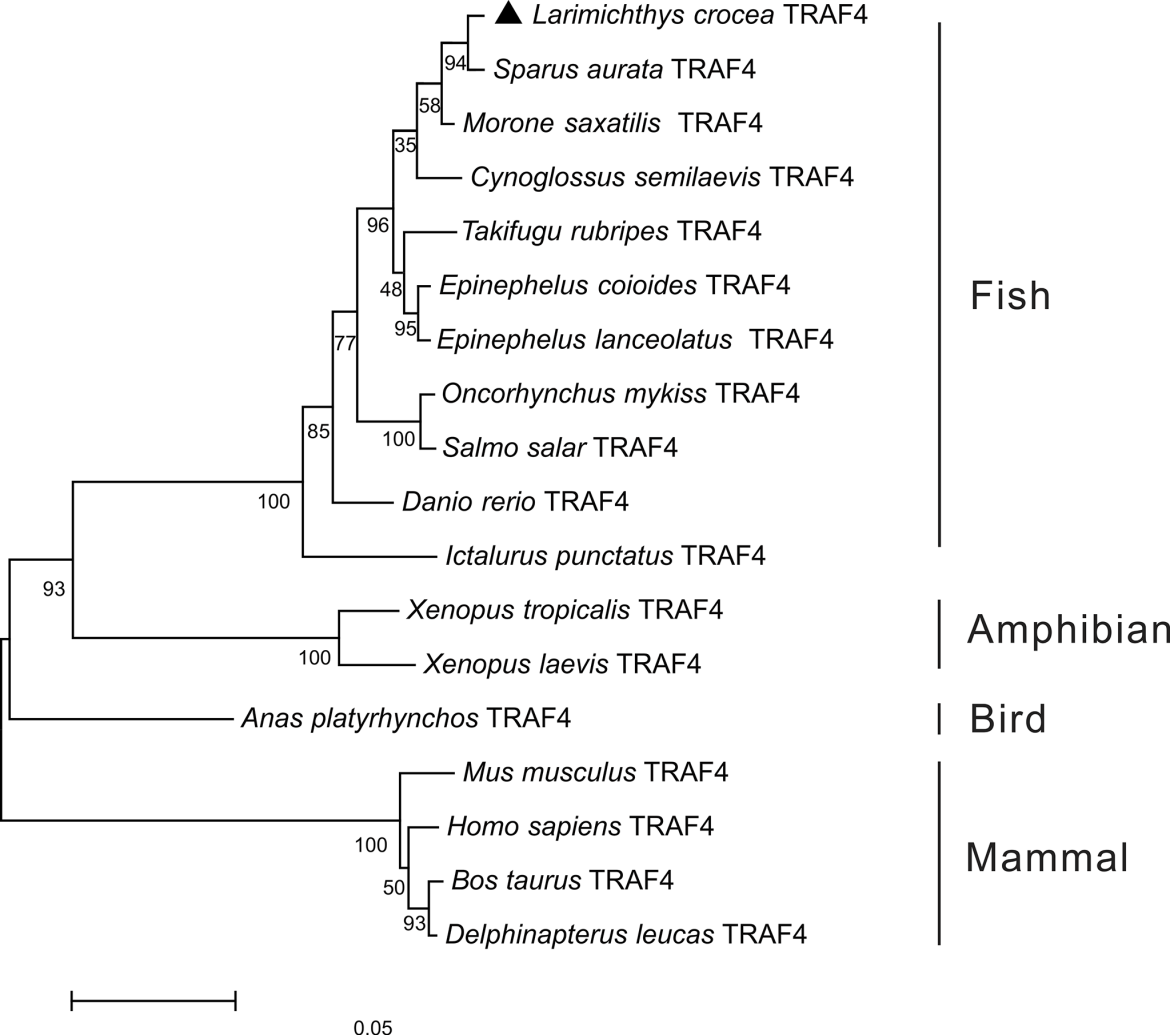
Phylogenetic analysis of vertebrate TRAF4. The phylogenetic tree was constructed based on the alignment of amino acid sequences of teleost, bird, amphibian and mammal TRAF4 using the neighbor-joining method by MEGA version 7.0 software with 10,000 replications of the bootstrap test. The number on the branch represents the percentage of the boot value. The GenBank association numbers of these sequences used are as follows: *L. crocea* TRAF4, ON246166; *Sparus aurata* TRAF4, XP_030259023.1; *Morone saxatilis* TRAF4, XP_035536590.1; *Cynoglossus semilaevis* TRAF4, XP_008307618.1; *T. rubripes* TRAF4, XP_003968588.1; *E. coioides* TRAF4, AME21333.1; *Epinephelus lanceolatus* TRAF4, XP_033488065.1; *O. mykiss* TRAF4, XP_021443644.1; *S. salar* TRAF4, NP_001167069.1; *D. rerio* TRAF4, NP_991325.1; *I. punctatus* TRAF4, XP_017346543.1; *Xenopus tropicalis* TRAF4, NP_001005074.1; *X. laevis* TRAF4, NP_001087501.1; *Anas platyrhynchos* TRAF4, XP_038021542; *Bos taurus* TRAF4, AAI51615.1; *M. musculus* TRAF4, NP_033449.2; *Delphinapterus leucas* TRAF4, XP_022451183.1; *H. sapiens* TRAF4, NP_004286.2.

To further understand the phylogenic relationship of TRAF4 in vertebrates, the phylogenetic tree was constructed based on the amino acid sequences of *Lc*-TRAF4 and other vertebrates, including teleosts, amphibians, birds, and mammals. The results showed that these TRAF4 orthologs were divided into two groups, with mammalian TRAF4 gathering in one clade, whereas teleost, bird, and amphibian TRAF4 gathering in another clade. Additionally, *Lc*-TRAF4 was clustered into fish TRAF4 family and was most closely related to TRAF4 of sea bream (*Sparus aurata*) (**Figure 1**).

### Genomic Organization of TRAF4 Genes in Vertebrates

**Figure 2:**
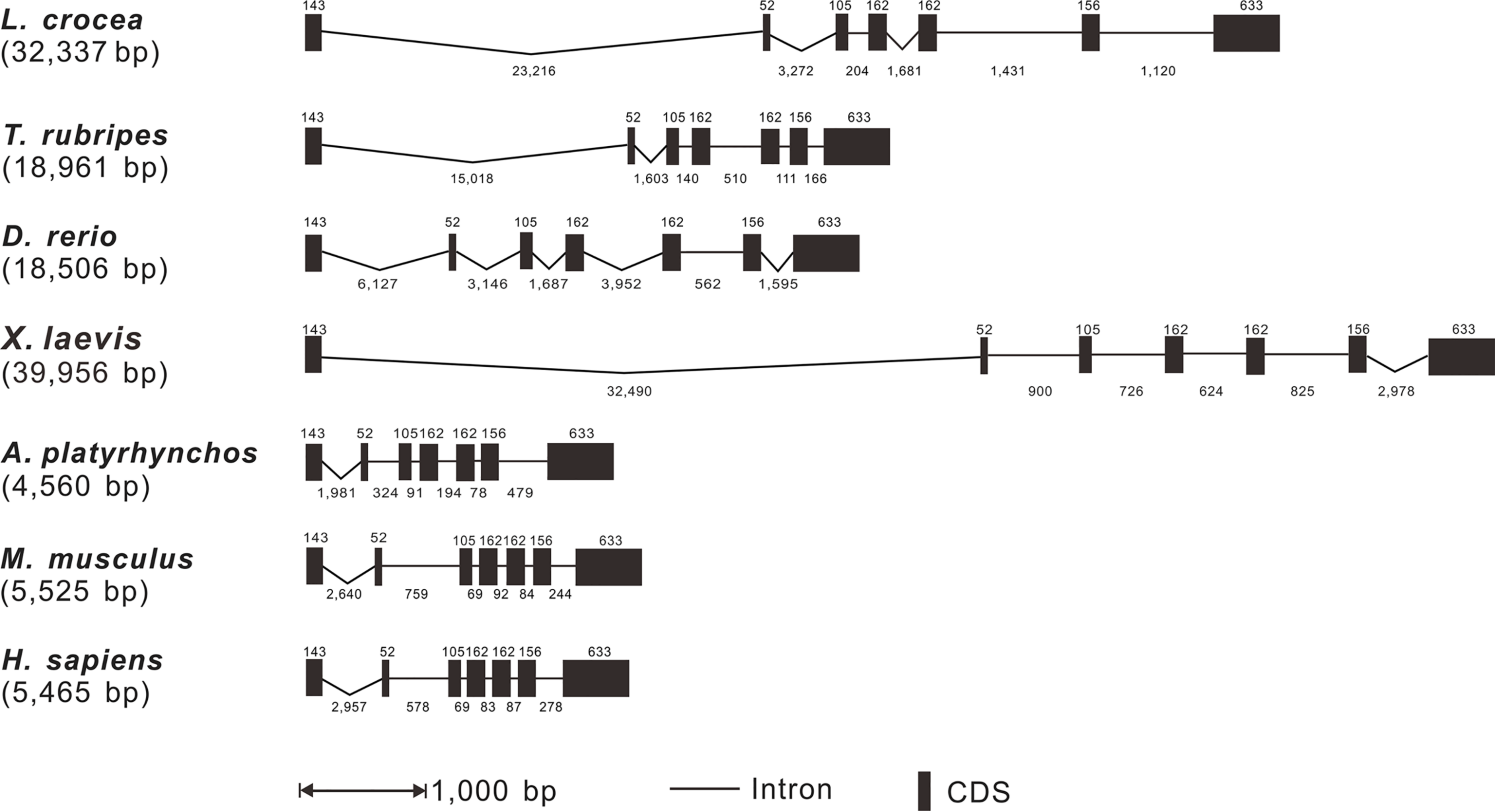
Genomic organization comparison of *Lc*-*TRAF4* with other vertebrates. Comparison of exons and introns of *TRAF4* gene in *L. crocea*, *T. rubripes*, *X. laevis*, *A. platyrhynchos*, *D. rerio*, *M. musculus* and *H. sapiens*. Exons and introns are represented by black boxes and lines, respectively, with the length of the exon shown above the black box and the length of the intron shown below the line. The length of the black box and the length of the line is proportional to the length of the sequence. Gene sequences information and their GenBank association numbers are shown as follows: *L. crocea*, NC_040017.1 (20102011-20136861); *T. rubripes*, NC_042295.1 (11474831-11495738); *D. rerio*, NC_007132.7 (39005909-39024739); *X. laevis*, NC_054374.1 (4177010-4219063); *A. platyrhynchos*, NC_051791.1 (5126618-5131704); *M. musculus*, NC_000077.7 (78049249-78056405); *H. sapiens*, NC_000017.11 (28744004-28750956).

Based on the analysis of genomic sequences of *Lc-TRAF4* and other vertebrate *TRAF4* genes in NCBI database, the genomic structures of large yellow croaker, fugu, zebrafish, African clawed frog, duck *(Anas platyrhynchos)*, mouse, and human were analyzed. The results showed that the length of *TRAF4* genes have a range of 4,560 bp to 39,956 bp from teleosts to mammals, with the shortest found in duck and the longest found in African clawed frog, respectively. In addition, the exon-intron organization of *TRAF4* in teleosts, amphibians, birds, and mammals was similar, including 7 exons and 6 introns (**Figure 2**). All exons of vertebrate *TRAF4* were very conserved, which were 143, 52, 105, 162, 162, 156, and 633 bp in length, respectively, whereas the sizes of introns were varied considerably in the detected species (**Figure 2**).

### Constitutive and Inductive Expression of *Lc*-TRAF4

**Figure 3:**
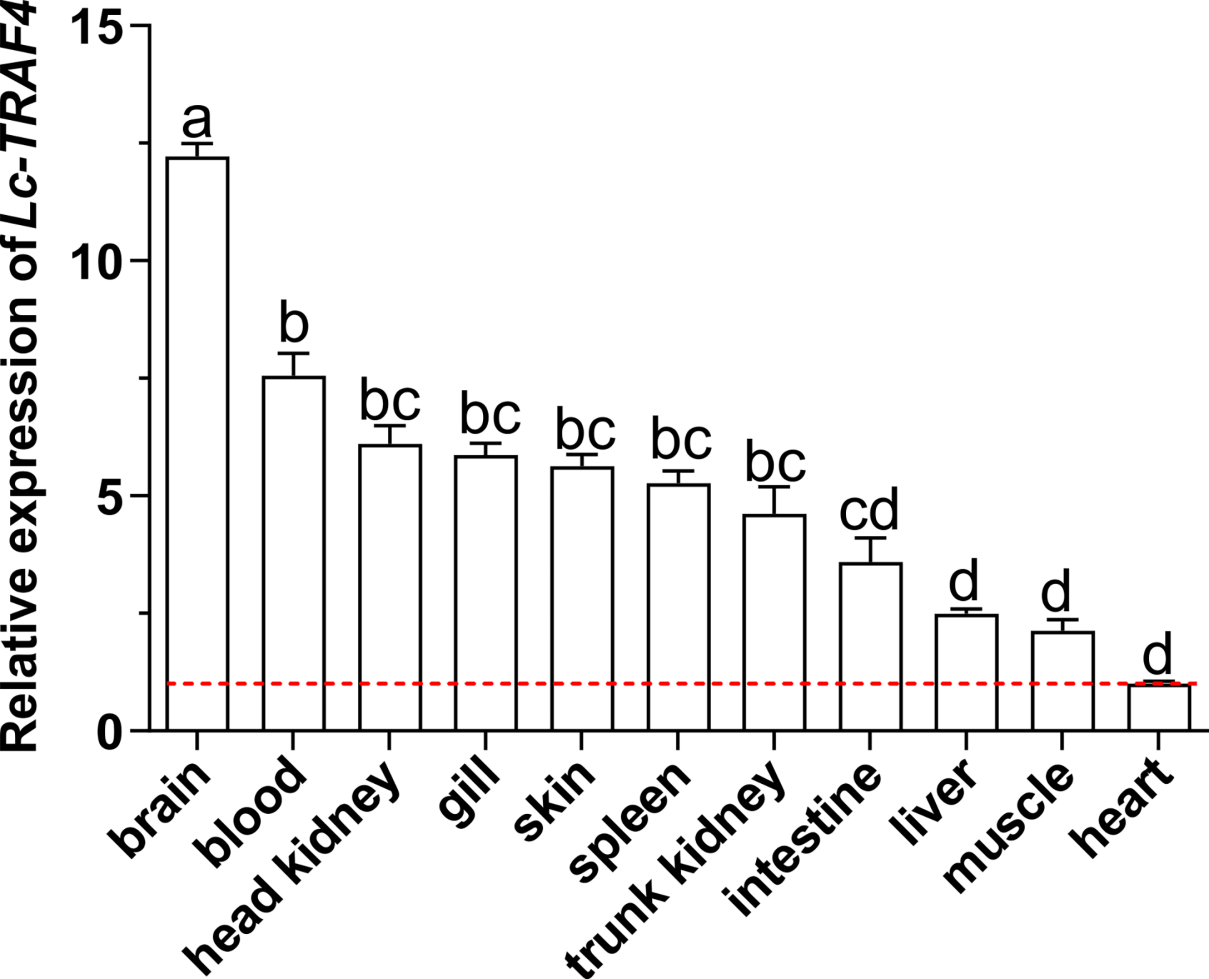
Distribution patterns of *Lc-TRAF4* mRNA in large yellow croaker. The expression patterns of *Lc-TRAF4* mRNA in 11 different tissues/organs of healthy large yellow croakers were detected by qRT-PCR analysis, with the results normalizing to the expression of *β-actin*. The mRNA expression level of *Lc-TRAF4* in the heart was set as 1-fold, and the mRNA expression levels of *Lc-TRAF4* in other tissues/organs were recorded as multiple of the expression level in the heart, and the baseline of 1-fold was marked with the red dotted line. All data were expressed as mean ± standard error (SE) (n = 6). Statistical analysis was performed using a one-way ANOVA followed by a Duncan’s multiple range test. Different superscripts indicate statistically different results (*P* < 0.05), and the same superscript indicates no statistical differences between groups.

The results of qRT-PCR analysis showed that *Lc-TRAF4* was ubiquitously expressed in all detected organs/tissues, including the gill, spleen, head kidney, trunk kidney, liver, heart, brain, skin, muscle, intestine, and blood, with the highest expression level found in the brain, and the lowest observed in the heart (**Figure 3**).

**Figure 4:**
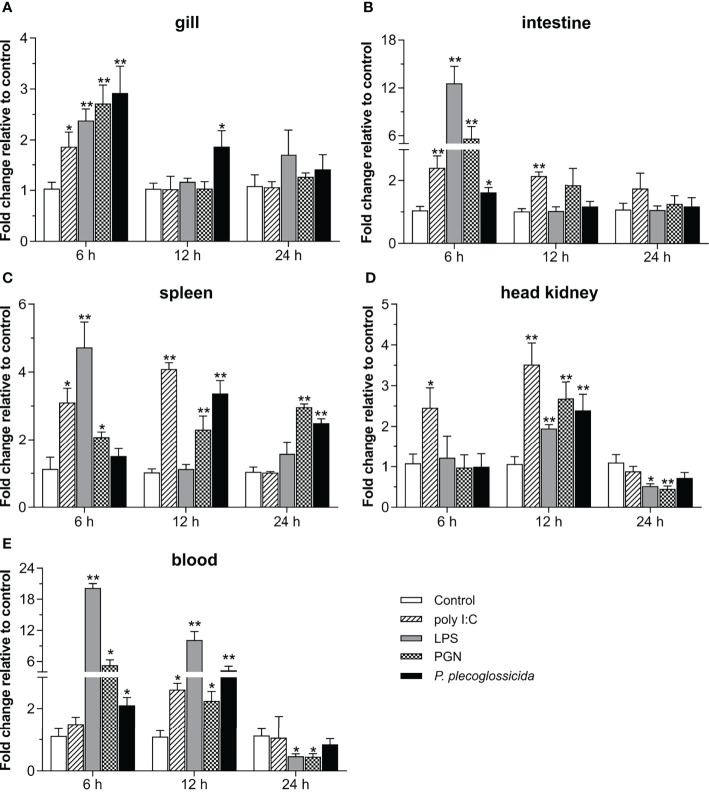
Expression patterns of *Lc-TRAF4* under poly I:C, LPS, PGN, and *P. plecoglossicida* stimulations. The healthy large yellow croakers were divided into five groups and then intraperitoneally injected with 100 μL of poly I:C (1 mg/mL), LPS (0.5 mg/mL), PGN (1 mg/mL), *P. plecoglossicida* suspension in PBS (5 × 10^5^ CFU/mL), and PBS (Control), respectively. Six large yellow croakers were randomly selected from each group at 6, 12 and 24 hpi, and the mRNA expression levels of *Lc-TRAF4* in the gill **(A)**, intestine **(B)**, spleen **(C)**, head kidney **(D)**, and blood **(E)** were detected by qRT-PCR analysis, respectively. The expression level changes were calculated by normalization to the expression of *β-actin* and then recorded as fold relative to the PBS injection group (control group) at the same sampling time. All data were expressed as mean ± SE (n = 6), with bars denoting SE. *P < 0.05, **P < 0.01.

To further understand the role of *Lc-TRAF4* in the host immune response, the expression patterns of *Lc-TRAF4* in mucosal immune tissues (gill and intestine), peripheral immune organ/tissue (spleen and head kidney), and peripheral blood under various PAMPs stimulations were also determined. The results showed that the expression levels of *Lc*-*TRAF4* were significantly induced under poly I:C stimulation in the gill, intestine, spleen, head kidney, and peripheral blood, with a 1.9-fold increase at 6 hpi in gill (**Figure 4A**), 2.4- and 2.1-fold, a 3.1- and 4.1-fold, and a 2.5- and 3.5-fold increase at 6 and 12 hpi in the intestine, spleen, and head kidney (**Figures 4B–D**), and a 2.6-fold increase at 12 hpi in peripheral blood (**Figure 4E**), respectively. The expression levels of *Lc*-*TRAF4* were also significantly up-regulated upon LPS challenge, which was a 2.4-, 12.6-, and 4.7-fold increase at 6 hpi in the gill, intestine, and spleen (**Figures 4A–C**), a 2.0-fold increase at 12 hpi in the head kidney (**Figure 4D**), and a 20.2- and 10.1-fold increase at 6 and 12 hpi in peripheral blood (**Figure 4E**), respectively. In addition, the expression levels of *Lc*-*TRAF4* were also significantly induced under PGN stimulation, with a 2.7- and 5.6-fold increase at 6 hpi in the gill and intestine (**Figures 4A,B**), a 2.1-, 2.3-, and 3.0-fold increase at 6, 12, and 24 hpi in the spleen (**Figure 4C**), a 2.7-fold increase at 12 hpi in the head kidney (**Figure 4D**), and a 5.3- and 2.2-fold increase at 6 and 12 hpi in peripheral blood (**Figure 4E**), respectively. Additionally, *Lc*-*TRAF4* was also significantly induced in response to *P. plecoglossicida* infection, with a 2.9- and 1.9-fold, a 2.1- and 4.3-fold increase at 6 and 12 hpi in gill and peripheral blood (**Figures 4A, E**), a 1.6-fold increase at 6 hpi in the intestine (**Figure 4B**), a 3.4- and 2.5-fold increase at 12 and 24 hpi in the spleen (**Figure 4C**), and a 2.4-fold increase at 12 hpi in the head kidney (**Figure 4D**), respectively. Nevertheless, the expression levels of *Lc-TRAF4* were significantly down-regulated at 24 hpi in the head kidney and peripheral blood under LPS and PGN stimulation (**Figures 4D, E**), respectively.

### *Lc*-TRAF4 Induces IRF3, IRF7, and Type I IFN Promoters Activation

**Figure 5:**
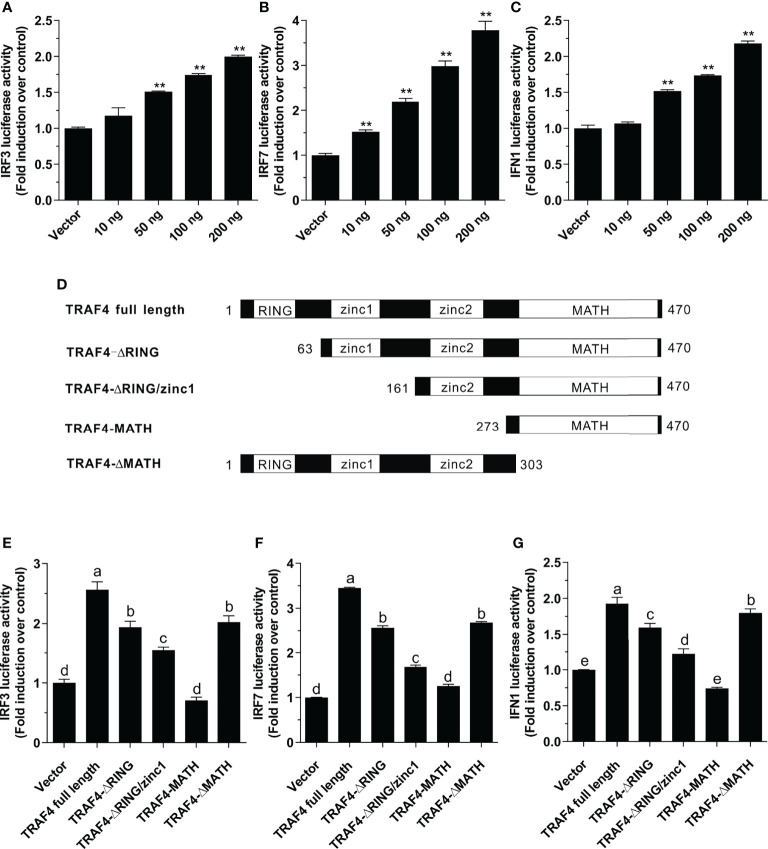
Function of *Lc*-TRAF4 and its truncated forms in IRF3, IRF7, and IFN1 promoter activation. **(A)** Induction of IRF3 promoter activity by *Lc*-TRAF4. **(B)** Induction of IRF7 promoter activity by *Lc*-TRAF4. **(C)** Induction of IFN1 promoter activity by *Lc*-TRAF4. HEK 293T cells seeded in 24-well plates were co-transfected with 100 ng of pGL4-IRF3-pro, pGL4-IRF7-pro or pGL4-IFN1-pro, 10 ng pRL-TK together with increasing amounts of pcDNA3.1-TRAF4 or pcDNA3.1 (vector control). At 24 hpt, the cells were collected to detect luciferase activity. **(D)** Schematic representation of *Lc*-TRAF4 full-length and the truncated forms used in this study. **(E)** Induction of IRF3 promoter activity by *Lc*-TRAF4 and its truncated forms. **(F)** Induction of IRF7 promoter activation by *Lc*-TRAF4 and its truncated forms. **(G)** Induction of IFN1 promoter activity by *Lc*-TRAF4 and its truncated forms. Briefly, HEK 293T cells seeded in 24-well plates were co-transfected with 100 ng of pGL4-IRF3-pro, pGL4-IRF7-pro or pGL4-IFN1-pro, 10 ng pRL-TK together with 100 ng of pcDNA3.1-TRAF4 or *Lc*-TRAF4 truncated forms expression plasmids including pcDNA3.1-TRAF4-ΔRING, pcDNA3.1-TRAF4-ΔRING/zinc1, pcDNA3.1-TRAF4-MATH, pcDNA3.1-TRAF4-ΔMATH or pcDNA3.1 empty vector as control. At 24 hpt, the cells were harvested for detection of luciferase activity. All data were shown as mean ± SE from three independent experiments, with bars representing SE. Different superscripts indicate statistically different results (P < 0.05), and the same superscript indicates no statistical differences between groups. *P < 0.05, **P < 0.01.

To investigate whether *Lc*-TRAF4 plays a role in IRF3, IRF7, and type I IFN promoter activation, expression plasmids pcDNA3.1-TRAF4 or pcDNA3.1 empty vector together with IRF3, IRF7, or IFN1 promoter luciferase reporter plasmids were co-transfected into HEK 293T cells and then detected by luciferase assays. The results showed the overexpression of *Lc*-TRAF4 could significantly induce the activation of IRF3, IRF7, and IFN1 promoters, which provoking a typical plasmid dose-dependent manner. With the transfection amount going higher, the induction level increased, being up to 2.0-, 3.8-, and 2.2-fold relative to the control at a plasmid dose of 200 ng in IRF3, IRF7, and IFN1 promoter activation, respectively (**Figures 5A–C**).

To determine the role of various domains of *Lc*-TRAF4 in IRF3, IRF7 or IFN1 promoter activation, four truncated forms of domains deletion expression vectors were constructed, including TRAF4-ΔRING (deletion of the RING finger domain), TRAF4-ΔRING/zinc1 (deletion of the RING finger and the first zinc finger domain), TRAF4-MATH (only containing the MATH domain), and TRAF4-ΔMATH (deletion of the MATH domain) (**Figure 5D**). Based on dual-luciferase reporter assays, it was found that the truncated forms of *Lc*-TRAF4, except for the only containing MATH domain mutant, can also effectively induce IRF3, IRF7, and IFN1 promoter activation. However, the promoter activation levels induced by truncated forms of *Lc*-TRAF4, including TRAF4-ΔRING, TRAF4-ΔRING/zinc 1, and TRAF4-ΔMATH were significantly lower compared to the full-length form of TRAF4 (**Figures 5E–G**). It is thus suggested that the RING finger and zinc finger domains of *Lc*-TRAF4 may function in the activation of IRF3, IRF7, and IFN1 signaling.

### Subcellular Localization of *Lc*-TRAF4

**Figure 6:**
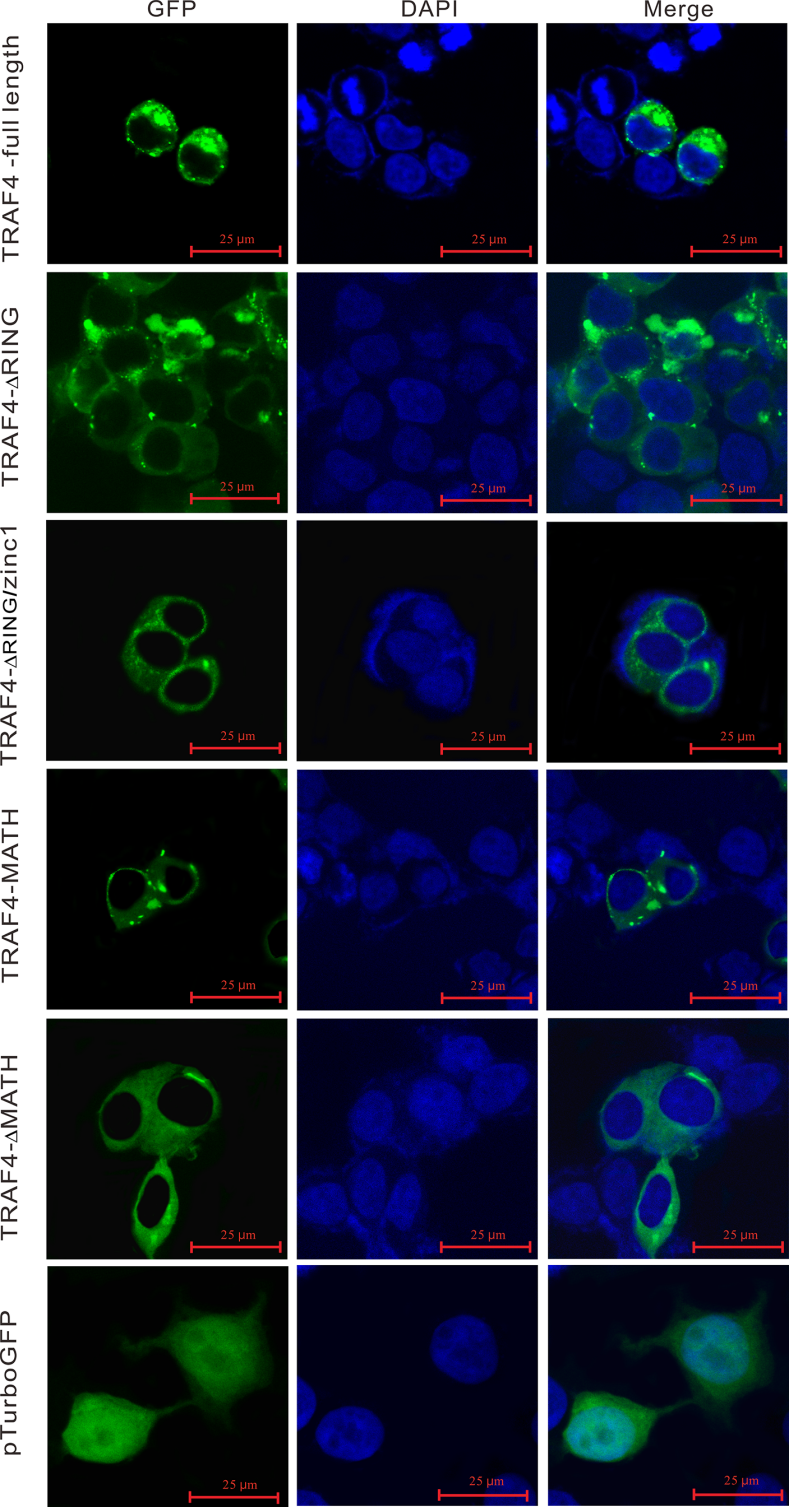
Subcellular localization of *Lc*-TRAF4 and its truncated forms. HEK 293T cells were transfected with pTurbo-TRAF4-GFP, pTurbo-TRAF4-ΔRING-GFP, pTurbo-TRAF4-ΔRING/zinc1-GFP, pTurbo-TRAF4-MATH-GFP, pTurbo-TRAF4-ΔMATH-GFP, and pTurboGFP-N (vector control), respectively. At 24 hpt, cells were stained with DAPI, detected and photographed under a confocal microscope.

In order to understand the subcellular localization of *Lc*-TRAF4, the full-length ORF of *Lc*-TRAF4 was subcloned into pTurboGFP-N vector and then transfected into HEK 293T cells, followed by examining under a confocal microscope at 24 hpt. The results showed that the pTurbo-TRAF4-GFP fusion protein was distributed in the cytoplasm, with bright green spots identified around the nucleus. To determine the function of various domains of *Lc*-TRAF4 in the subcellular distribution, four truncated forms of domain deletion expression vectors, including TRAF4-ΔRING, TRAF4-ΔRING/zinc1, TRAF4-MATH, and TRAF4-ΔMATH were also transfected into HEK 293T cells and assayed. It was revealed that TRAF4-ΔRING, TRAF4-ΔRING/zinc1, TRAF4-MATH, and TRAF4-ΔMATH had nearly the same subcellular distribution with the full length of *Lc*-TRAF4, which were also located in the cytoplasm with some aggregates around the nucleus. However, the control cells transfected with an empty pTurboGFP-N vector showed a global cytosolic localization as well as the nucleus (**Figure 6**).

### *Lc*-TRAF4 Associates With *Lc*-TRIF and *Lc*-TRAF6 in Antiviral and Inflammatory Signaling

**Figure 7:**
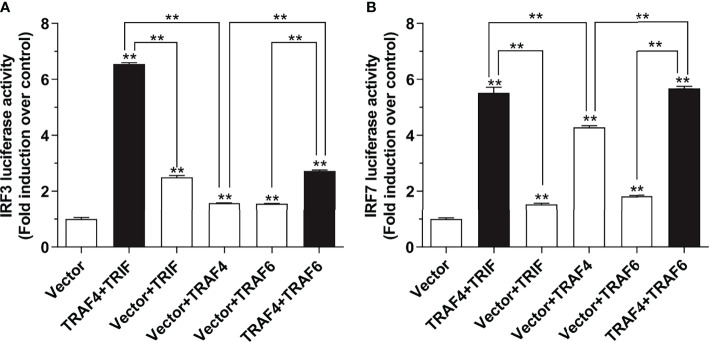
*Lc*-TRAF4 associated with *Lc*-TRIF and *Lc*-TRAF6 in IRF3 and IRF7 promoter activation. **(A)** The association of *Lc*-TRAF4 with *Lc*-TRIF and *Lc*-TRAF6 in IRF3 promoter activation. **(B)** The association of *Lc*-TRAF4 with *Lc*-TRIF and *Lc*-TRAF6 in IRF7 promoter activation. HEK 293T cells were co-transfected with 100 ng of pGL4-IRF3-pro or pGL4-IRF7-pro, 10 ng of pRL-TK together with 100 ng of pcDNA3.1-TRAF4, pcDNA3.1-TRIF and pcDNA3.1-TRAF6 alone or in a combination of two. The pcDNA3.1 empty vector was added to balance the total amounts of transfected plasmids between different wells. At 24 hpt, the cells were harvested for the detection of luciferase activities. All data were presented as mean ± SE from three independent experiments, with bars representing SE. *P < 0.05, **P < 0.01.

As we have mentioned above, *Lc*-TRAF4 overexpression was sufficient to activate the promoter activity of IRF3 and IRF7, which are involved in the host antiviral signaling pathway. It is thus important to reveal the molecules that associated with *Lc*-TRAF4 in such signaling. To address this question, expressing plasmids including *Lc*-TRAF4, *Lc*-TRIF, and *Lc*-TRAF6 were co-transfected with IRF3 or IRF7 promoter reporter plasmids into HEK 293T cells and then detected by dual-luciferase reporting system. The results showed that co-expression of *Lc*-TRAF4 with *Lc*-TRIF could induce higher activation of IRF3 and IRF7 promoter compared with the only overexpression of *Lc*-TRAF4 or *Lc*-TRIF (**Figures 7A, B**). Additionally, co-transfection of *Lc*-TRAF4 with *Lc*-TRAF6 could also remarkably up-regulated the level of IRF3 and IRF7 promoter activation in comparison with the only transfection of *Lc*-TRAF4 or *Lc*-TRAF6 (**Figures 7A, B**). These results indicated that *Lc*-TRAF4 is associated with *Lc*-TRIF and *Lc*-TRAF6 in the activation of IRF3 and IRF7 signaling.

**Figure 8:**
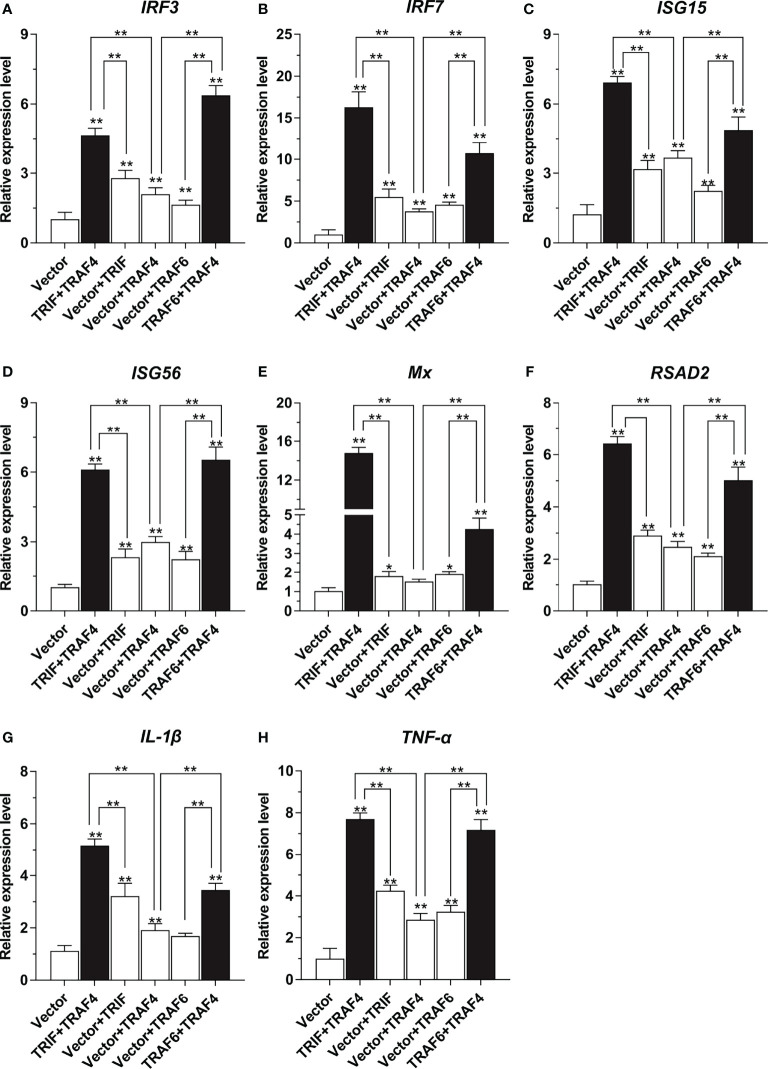
Association of *Lc*-TRAF4 with *Lc*-TRIF and *Lc*-TRAF6 in the induction of antiviral and inflammation-related genes expression. LYCMS cells were co-transfected with pcDNA3.1-TRAF4, pcDNA3.1-TRIF, and pcDNA3.1-TRAF6 alone or in a combination of two, with the total amounts of the transfected plasmids balanced by the pcDNA3.1 empty vector for each transfection. The cells were then collected for total RNA extraction and cDNA synthesis at 48 hpt. The mRNA expression levels of antiviral and inflammation-related genes, including *IRF3* **(A)**, *IRF7* **(B)**, *ISG15* **(C)**, *ISG56* **(D)**, *Mx* **(E)**, *RSAD2*(F), *IL-1β* **(G)**, and *TNF-α* **(H)** were examined by qRT-PCR, with the results calculated by the normalization to the expression of *β-actin* and then recorded as a fold change compared to the control group. All of the data were shown as mean ± SE from three independent experiments, with bars representing SE. *P < 0.05, **P < 0.01.

To further confirm the association of *Lc*-TRAF4 with *Lc*-TRIF and *Lc*-TRAF6 in the host immune-related signaling pathway, qRT-PCR analysis was also performed to reveal the association of *Lc*-TRAF4 with *Lc*-TRIF and *Lc*-TRAF6 on the regulation of antiviral as well as inflammation-related genes expression, including *IRF3, IRF7, ISG15, ISG56, Mx, RSAD2*, *IL-1β*, and *TNF-α*. The results showed that the mRNA expression levels of some immune-related genes described above were significantly induced under the overexpression of *Lc*-TRAF4 alone, including *IRF3, IRF7, ISG15, ISG56, RSAD2*, *IL-1β*, and *TNF-α*, whereas the expression of *Mx* was not affected (**Figures 8A–H**). Interestingly, co-expression of *Lc*-TRAF4 with *Lc*-TRIF and *Lc*-TRAF6 resulted in significantly higher expression levels of the detected immune-related genes compared with the only overexpression of *Lc*-TRAF4, *Lc*-TRIF or *Lc*-TRAF6 (**Figures 8A–H**), indicating that *Lc*-TRAF4 strongly associate with *Lc*-TRIF and *Lc*-TRAF6 in in induction of host antiviral and inflammatory signaling.

## Discussion

TRAF4 was originally named as CART1 because it contains a conserved cysteine-rich domain associated with RING and TRAF domains, which was first identified in metastatic lymph node tissue in *Homo sapiens* (31). Compared to the extensive and in-depth studies of other members of the TRAF family, such as TRAF3 and TRAF6, the function of teleost TRAF4 in host immune response remain largely unknown (8, 21, 32). Notably, the present study for the first time demonstrated that teleost TRAF4 could significantly induce IRF3, IRF7, as well as type I IFN promoter activation, and the associations of TRAF4 with TRIF and TRAF6 in inducing IRF3 and IRF7 promoter activation as well as antiviral and inflammation-related genes expression were also determined, which revealed the important regulatory function of teleost TRAF4 that involved in host immune responses.

Tissue expression analysis showed that *Lc*-*TRAF4* was widely expressed in different tissues/organs of large yellow croaker, with the highest expression level in the brain and the lowest expression level in the heart. The expression analysis of *TRAF4* in other teleosts showed that the expression level of *TRAF4* was the highest in the gill of Chinese tongue sole (21) and skin of grouper (19), respectively. These results suggest that the tissue expression patterns of *TRAF4* in teleosts may vary from different species. In addition, the expression level of *Lc*-*TRAF4* was up-regulated by poly I:C, LPS, PGN, and *P. plecoglossicida* stimulations in gill, intestine, spleen, head kidney, and blood, which were similar to studies of other teleosts that *TRAF4* in Chinese tongue sole could be induced under *Vibrio harveyi* infection (21), and grouper *TRAF4* could be activated in response to *Cryptocaryon irritans* infection (19). It is thus speculated that TRAF4 functions in response to bacterial and viral infections in teleosts.

IRF3 and IRF7 are key regulators of type I IFNs production and function in host antiviral immune responses (33). Interestingly, the present study revealed for the first time that teleost TRAF4 could significantly induce the activation of IRF3, IRF7 and also type I IFN promoters, and such induction was in a dose-dependent manner, suggesting that *Lc*-TRAF4 function in host immune response *via* IRF3 and IRF7 mediated signaling pathway. It was also found that although the truncated forms of *Lc*-TRAF4 including TRAF4-ΔRING, TRAF4-ΔRING/zinc 1, and TRAF4-ΔMATH could significantly induce IRF3, IRF7, and IFN1 promoter activation, the induction levels of such promoters were significantly lower compared to the full length of *Lc*-TRAF4. In addition, the mutant containing only the MATH domain exhibited no function in IRF3, IRF7, or IFN1 promoter activation. Studies in other TRAF members in teleosts also revealed that the deletion of grouper TRAF5 RING finger or zinc finger domain significantly impaired NF-κB activation (34), and overexpression of Nile tilapia TRAF5 could activate NF-κB, whereas deletion of RING finger or zinc finger domain caused impaired NF-κB activity (35). Combined with these data, it is thus demonstrated that the RING finger and zinc finger domains of the TRAF family function importantly in that mediated signaling.

It has been well documented that TRIF is essential for TLR3 and TLR4-mediated production of type I IFNs and other proinflammatory mediators in mammals (36). Similarly, TRIF has been identified as an important adaptor of TLR3, TLR19, and TLR22 mediated signaling pathways and plays important roles in immune responses against bacterial and viral infections in teleosts (37–39). Our previous results also revealed that large yellow croaker TRIF overexpression could induce NF-κB, IRF3, IRF7, and type I IFN promoter activation (27). Notably, the results of the present study showed that co-expression of *Lc*-TRAF4 with *Lc*-TRIF significantly enhances IRF3 and IRF7 promoter activation compared with the only overexpression of *Lc*-TRAF4 or *Lc*-TRIF. Additionally, the co-expression of *Lc*-TRAF4 with *Lc*-TRIF could significantly induce the expression levels of antiviral and inflammation-related genes compared to overexpression of *Lc*-TRAF4 or *Lc*-TRIF alone, such immune-related genes, including *IRF3*, *IRF7*, *ISG15*, *ISG56*, *Mx*, *RSAD2*, *IL-1β*, and *TNF-α*. It is thus collectively suggesting the association of *Lc*-TRAF4 with *Lc*-TRIF in host immune response, and *Lc*-TRAF4 may function as an enhancer in *Lc*-TRIF mediated signaling. Studies in mammals also reported the association of TRAF4 with TRIF, in which mammalian TRAF4 interacts with TRIF but inhibits TRIF mediated NF-κB and IFN-β promoter activation in TLR signaling (17), implying the functional differences of TRAF4 in teleosts from that in mammals, and that differences may result from the species differences.

TRAF6, an important member of the TRAF family, acts as an adaptor protein involved in TLRs, NLRs, and RLRs mediate the signaling pathway and plays important roles in host innate and adaptive immunity (8, 40). Mammalian TRAF6 has been reported to be involved in TRIF-mediated type I IFN and NF-κB activation (8), studies in teleosts revealed that black carp TRAF6 overexpression could significantly enhance TRIF mediated IFN activation (41), and our previously published study in large yellow croaker also found that *Lc*-TRAF6 overexpression significantly enhanced *Lc*-RIP1 mediated NF-κB activation (30). These results collectively suggested that TRAF6 plays important roles in the host immune signaling pathway. Interestingly, our results in the present study revealed that *Lc*-TRAF4 could associate with *Lc*-TRAF6 in the induction of IRF3 and IRF7 promoter activation, and co-expression of *Lc*-TRAF4 with *Lc*-TRAF6 also significantly up-regulated the expression levels of antiviral and inflammation-related genes including *IRF3*, *IRF7*, *ISG15*, *ISG56*, *Mx*, *RSAD2*, *IL-1β*, and *TNF-α* compared to the only overexpression of *Lc*-TRAF4 or *Lc*-TRAF6, collectively indicating the association of *Lc*-TRAF4 with *Lc*-TRAF6 in host immune signaling, especially in IRF3 and IRF7 mediated signaling pathway. Notably, studies in mammals revealed that TRAF4 physically interacts with TRAF6 but suppresses TRAF6 mediated NF-κB and IFN-β promoter activation (17), and mammalian TRAF4 also exerts its negative regulation on IL-17 signaling by competing with TRAF6 for the interaction with Act1 (18). It is thus suggested that although teleost TRAF4 associates with TRAF6 in the host immune signaling, the functionally regulation is not the same as that found in mammals, which function as an enhancer but not a silencer in TRAF6 mediated signaling in teleosts.

In conclusion, the present study identified a TRAF4 ortholog in large yellow croaker, named *Lc*-*TRAF4*, which could be up-regulated in response to stimulations of poly I:C, LPS, PGN, and *P. plecoglossicida.* As a cytoplasmic protein, *Lc*-TRAF4 could significantly induce IRF3, IRF7, as well as type I IFN promoter activation. Notably, overexpression of *Lc*-TRAF4 significantly enhanced *Lc*-TRIF and *Lc*-TRAF6 mediated IRF3 and IRF7 promoter activation, and also the expression level of antiviral and inflammation-related genes. It is collectively indicated that *Lc*-TRAF4 function as an enhancer in *Lc*-TRIF and *Lc*-TRAF6 mediated immune signaling, which shed new light on understanding the diversity function of TRAF4 in vertebrates.

## Data Availability Statement

The original contributions presented in the study are included in the article/**Supplementary Material**. Further inquiries can be directed to the corresponding authors.

## Ethics Statement

The animal study was reviewed and approved by Animal Administration and Ethics Committee of Jimei University (Permit No. 2021-4).

## Author Contributions

PZ conceived and designed the research. PZ, YC, YL, PL, and ZL performed the experiments and analyzed the data. PZ, YC, and YL wrote the manuscript. PZ, YL, ZZ, and YW revised the manuscript. All authors have read and agreed to the published version of the manuscript.

## Funding

This work was supported by a grant (31772878) from the National Natural Science Foundation of China, a grant (2021J02046) from the Natural Science Foundation of Fujian Province of China, and a grant (3502Z20206017) from the Youth Innovation Foundation of Xiamen City of China.

## Conflict of Interest

Authors ZPZ and YLW are honorary scientists of the Ningde Fufa Fisheries Company Limited. 

The remaining authors declare that the research was conducted in the absence of any commercial or financial relationships that could be construed as a potential conflict of interest.

## Publisher’s Note

All claims expressed in this article are solely those of the authors and do not necessarily represent those of their affiliated organizations, or those of the publisher, the editors and the reviewers. Any product that may be evaluated in this article, or claim that may be made by its manufacturer, is not guaranteed or endorsed by the publisher.

## Supplementary Material

Supplementary Figure 1 Multiple alignment of the protein sequence of *Lc*-TRAF4 with other vertebrate TRAF4. Black shaded sequences indicate locations where all sequences share the same amino acid residues, whereas gray shading and light gray shading represent conserved and semi-conserved amino acid substitutions, respectively. The conservation RING finger domain, zinc finger domain, and MATH domain are represented with black arrows, respectively.
